# Disparities in Gynecological Malignancies

**DOI:** 10.3389/fonc.2016.00036

**Published:** 2016-02-22

**Authors:** Sudeshna Chatterjee, Divya Gupta, Thomas A. Caputo, Kevin Holcomb

**Affiliations:** ^1^Division of Gynecologic Oncology, Department of Obstetrics and Gynecology, Weill Cornell Medical College, New York Presbyterian Hospital, New York, NY, USA

**Keywords:** heath disparities, gynecologic malignancies, race, socioeconomic factors, barriers to health

## Abstract

**Objectives:**

Health disparities and inequalities in access to care among different socioeconomic, ethnic, and racial groups have been well documented in the U.S. healthcare system. In this review, we aimed to provide an overview of barriers to care contributing to health disparities in gynecological oncology management and to describe site-specific disparities in gynecologic care for endometrial, ovarian, and cervical cancer.

**Methods:**

We performed a literature review of peer-reviewed academic and governmental publications focusing on disparities in gynecological care in the United States by searching PubMed and Google Scholar electronic databases.

**Results:**

There are multiple important underlying issues that may contribute to the disparities in gynecological oncology management in the United States, namely geographic access and hospital-based discrepancies, research-based discrepancies, influence of socioeconomic and health insurance status, and finally the influence of race and biological factors. Despite the reduction in overall cancer-related deaths since the 1990s, the 5-year survival for Black women is significantly lower than for White women for each gynecologic cancer type and each stage of diagnosis. For ovarian and endometrial cancer, black patients are less likely to receive treatment consistent with evidence-based guidelines and have worse survival outcomes even after accounting for stage and comorbidities. For cervical and endometrial cancer, the mortality rate for black women remains twice that of White women.

**Conclusion:**

Health care disparities in the incidence and outcome of gynecologic cancers are complex and involve biologic factors as well as racial, socioeconomic, and geographic barriers that influence treatment and survival. These barriers must be addressed to provide optimal care to women in the U.S. with gynecologic cancer.

## Introduction

The Institute of Medicine released a landmark report in 2003 titled “Unequal Treatment: Confronting Racial and Ethnic Disparities in Health Care,” which demonstrated disparities in the U.S. health care system between treatment of racial and ethnic minorities and Whites, subsequently resulting in poorer health outcomes for millions of Americans (1).

Since that time, the National Cancer Institute (NCI) through the Center to Reduce Cancer Health Disparities (CRCHD), the American Cancer Society, the American Society of Clinical Oncology, and the Society of Gynecologic Oncology has committed to the goals of eliminating disparities in cancer-related outcomes (2–4). The NCI defines cancer health disparities as “differences in the incidence, prevalence, mortality, and burden of cancer and adverse related conditions that exist among specific population groups in the United States (2).”

The etiology of disparities in cancer treatment and outcomes has been linked to the complex interplay of race/ethnicity, cultural, socioeconomic, and educational factors. Geographic variability in provider and hospital standards and biological differences between ethnic groups must also be considered (1, 4, 5). Finally, variation from evidenced-based treatment guidelines has been indicated as a modifiable factor that can result in poorer survival outcomes (6).

This review aims to describe some of the important underlying issues that may contribute to the disparities in gynecological oncology management in the United States, namely geographic access and hospital-based discrepancies, research-based discrepancies, influence of socioeconomic and health insurance status, and finally the influence of race. This review continues with detailing site-specific disparities in gynecologic care for endometrial, ovarian, and cervical cancer.

## Geographic Access and Hospital-Based Discrepancies

A growing body of evidence has demonstrated the importance of access to high-volume hospitals and providers for optimal management and outcomes related to gynecologic malignancies. Several studies have demonstrated worse survival outcomes associated with low volume hospital centers and providers (6–9). A recent analysis of 96,000 patients with ovarian cancer identified by the National Cancer Data Base demonstrated 56% of patients were not receiving the standard of care as designated by the National Comprehensive Cancer Network (NCCN) guidelines. The study also demonstrated that 25% of women received care at very low volume institutions, defined as those treating one to seven cases of ovarian cancer annually. The authors concluded that deviation from NCCN guidelines and treatment at very low volume institutions were both independent predictors of worse disease-specific overall survival (hazard ratio 1.33, 95% CI 1.26–1.41 and HR 1.08, 95% CI 1.01–1.16, respectively) (6, 7). A prior study had also demonstrated that lower volume centers were less likely to provide recommended comprehensive surgical staging procedures (10). An analysis of Surveillance, Epidemiology, End Results (SEER) database demonstrated that chemotherapeutic treatments also varied depending on geography and available oncological providers (11, 12). Per the US Census Bureau, 81% of people live in cities or suburbs with 19% living in rural areas. Shalowitz et al. recently reported that an estimate 7663 women with gynecological malignancies (9% of the total cases of gynecological cancers per year) live in low-access counties in the US located 50 miles from the nearest gynecologic oncologist. These counties were more likely be rural, have residents with lower median incomes, and have more White and Hispanic patients than counties in closer proximity to gynecologic oncologists (13). Although this study did not include outcomes data, prior studies have reported that treatment by a trained gynecological oncologist with increased operative volume yields favorable survival outcomes (14–17). Other studies also associated increasing distance from a gynecological oncologist with increased cervical and endometrial cancer mortality (18). It is therefore important to consider geographic and hospital system-related disparities which influence both access to care and adherence to evidence-based treatment guidelines.

## Research-Based Discrepancies

Given varied survival outcomes among minority patients, there has been increased focus on attempting to recruit minorities for clinical trials to elucidate inherent differences in tumor biology, response to therapy, and survival in clinical situations where treatment regimens are controlled between groups. The National Institutes of Health (NIH) Revitalization Act of 1993 specifically addressed this issue encouraging enrollment of women and minorities to NIH-sponsored research. However, upon analysis of the four most common NCI-funded clinical trials (breast, prostate, colorectal, and lung cancers) from 1996 through 2002, investigators found that although clinical trial enrollment rate increased by almost 50% during this time period, the proportion of trial participants who were non-White actually declined – Hispanic patients from 3.7% of trial participants to 3.0% and Black patients from 11.0% of trial participants to 7.9% (19). It is not surprising that decreased minority enrollment in clinical trial also exists for gynecologic cancers. Scalici et al. recently published their paper on minority participation in 170 Gynecologic Oncology Group (GOG) trials from 1994 to 2013. They reported that of a total of 45,259 patients were included in GOG trials with 83% being White, 8% Black, and 9% other (*p* < 0.01). They also used Center for Disease Control (CDC) age-adjusted incidence to determine that observed enrollment of Black patients was 15 times lower than expected for ovarian cancer trials, 10 times lower than expected for endometrial cancer trials, 4.5 times lower than expected for cervical cancer trials, and 5.2 times lower than expected for sarcoma trials (*p* < 0.001) irrespective of the type of study or year published (20). Scalici et al. also found that African American participation in clinical trials actually decreased from 16% from 1994 to 2002 to 6% from 2009 to 2013. A recent review utilizing qualitative interviews concluded that the key barriers to minority recruitment to clinical trials were lack of opportunities to participate and lack of encouragement to enroll (21). Additionally, language barriers and logistical issues such as cost of travel may play a role in the recruitment of some minority populations (22). Prior studies have implicated a reduced acceptance to enrollment due to minority skepticism as a factor for reduced involvement in clinical trials (21, 23, 24). However, a study evaluating clinical trial consent rates by race demonstrated no difference in the willingness of Blacks and Hispanics to participate in health research compared to non-Hispanic Whites when offered clinical trial enrollment (25). To fully understand and optimize treatment for minority patients, it is imperative that these issues be addressed. The NRG Oncology Accrual Workshop recently held a meeting to increase minority recruitment for clinical trials (26).

## Socioeconomic Status and Health Insurance Status

Individuals with lower socioeconomic status have disproportionately higher cancer incidence rates and death rates than those with higher socioeconomic status, regardless of demographic factors such as race/ethnicity (27). According to the US Census Bureau’s report on Income and Poverty for 2014, the median household income in the US was $53,657 with significant variation by race, with Asians the highest at $74,297, and Blacks the lowest at $35,398 (28). The official poverty rate was 14.8%, accounting for 46.7 million people. In 2014, women made an average 79% of what men earned with a median income of $39,621 compared to $50,383 earned by men. Sixteen percent of women were below the poverty line, compared to 13.4% of males. Gender differences in poverty rates were more pronounced for those aged 65 and older (12.1% for women vs. 7.4% for men). Ten percent of non-Hispanic Whites, 12.0% of Asians, 26.2% of Blacks, and 23.6% of Hispanics lived below the poverty level (29). The US Census Bureau’s report of Health Insurance Coverage in the US reported the percentage of people without health insurance coverage decreased by 10.4%, or 33.0 million in 2013, compared to the number of uninsured in 2013. Despite these great strides in providing health insurance in the US, Blacks and Hispanics still have a higher rate of uninsured individuals compared to Asians and non-Hispanic Whites (11.8 and 19.9% vs. 9.3 and 7.6%, respectively). Additionally, 16.6% of uninsured individuals earned <$25,000 per year (30). Individuals in lower socioeconomic groups often present with advanced stage disease and are less likely to receive standard regimens of treatment (31).

A recent study by Bristow et al. evaluating the SEER-Medicare database for advanced ovarian cancer found poorer adherence to NCCN treatment guidelines associated with low socioeconomic status [OR 1.32, 95% CI (1.14–1.52)] and worse survival when accounting for the effects of other variables [HR 1.25, 95% CI (1.17–1.34)] despite equivalent Medicare insurance status (32). Additionally, insurance status seems to affect the type of care provided. Goff et al. demonstrated that payer status (private insurance vs. Medicaid) significantly impacted the chance of undergoing optimal surgical management in ovarian cancer (14). Esselen et al. demonstrated that Black women and uninsured women/women with Medicaid were less likely to undergo minimally invasive hysterectomies for uterine or cervical cancer after analysis of 46,450 women identified by the National Inpatient Sample (33). In a previous study by Harlan et al. examining 11 different cancer types, investigators noted significantly lower adherence to treatment guidelines for Black patients with Medicaid compared to Black patients with Medicare or private insurance (27). The same investigators found lack of private insurance a barrier to guideline based treatment for Black and Hispanic women with ovarian cancer, suggesting health insurance status may serve as proxy for other socioeconomic factors (34). Similarly, another analysis of adherence to NCCN guidelines in patients with ovarian cancer identified through the National Cancer Data Base demonstrated median household income of less than $35,000 was associated with non-adherence to evidence-based guidelines (OR 1.26, 95% CI 1.21–1.32) and worse survival (HR 1.06, 95% CI 1.02–1.1) (35). These findings were consistent with prior studies that have linked poverty level and low socioeconomic status to poorer adherence to evidence-based treatments and worse ovarian cancer survival (36–38). In addition to treatment administration, a recent evaluation of 8211 elderly patients with ovarian cancer identified from the SEER database demonstrated a decreased chance of hospice referral associated with non-White race [OR 1.44; 95% CI (1.26–1.65), *p* < 0.001], the lowest income group [OR 1.17; 95% CI (1.04–1.32), *p* = 0.01], and Medicare fee-for-service (vs. managed care) [OR 1.39; 95% CI (1.24–1.56), *p* < 0.001] (39).

## Race

Per the US Census Bureau, as of 2015, there are 321,729,000 people living in the United States with approximately 63.7% of the population described as Non-Hispanic White, 16.4% of the population described as Hispanic or Latino, 12.2% of the population described as African American, 4.7% Asian, and 0.9% Native American, Hawaiian, or Alaskan Native. Black men and women are more likely to die from cancer than any racial or ethnic group (31). Despite the reduction in overall cancer-related deaths since the 1990s, the 5-year survival for Black women is significantly lower than for White women at each stage of diagnosis, with the gap in survival actually increasing over the past few decades (40). Although the incidence of a new cancer diagnosis per 100,000 individuals is lower for Black women than White women (391.7 vs. 418.3, OR 0.94, *p* < 0.05), the death rate per 100,000 individuals is higher (180.6 for Blacks vs. 155.0 for Whites, OR 1.17, *p* < 0.05) (40). Interestingly, for all cancer sites, Hispanic women had a lower incidence of cancer relative to non-Hispanic White women [333.2 per 100,000 individuals compared to 433.9 per 100,000 (RR 0.8, *p* < 0.05)]. Additionally, for all cancer sites, Hispanic women had a favorable prognosis compared to non-Hispanic women with a mortality rate of 100.5 per 100,000 compared to 154.7 per 100,000 (RR 0.6, *p* < 0.05) (41). A notable exception is cervical cancer, where the incidence per 100,000 individuals for Hispanics was 11.8, compared to 7.2 for non-Hispanic Whites (RR 1.6, *p* < 0.05) and the mortality rate was 3.0 per 100,000 for Hispanics and 2.1 per 100,000 non-Hispanic Whites (RR 1.5, *p* < 0.05) (41). In general, Asian women had lower incidence and mortality rates than non-Hispanic White women across all cancer types (42–44). Among all Asians, the incidence of cancer per 100,000 is 314.9 with a mortality rate of 115.5 per 100,000, which is notably lower than that for non-Hispanic Whites (477.5 and 190.7, RR 0.7 and RR 0.6, *p* < 0.05, respectively) (45).

## Ovarian Cancer

Epithelial ovarian cancer (EOC) is the fifth cause of cancer death among women in the United States, accounting for an estimated 21,290 new cases and 14,180 cancer deaths in the US in 2015 (31). With aggressive surgical and chemotherapeutic management, overall survival has improved from 36% during the period of 1975–1977 to 45% during the period 2004–2010 (*p* < 0.05). However, the survival rate over the same time period for Black women has actually decreased from 42 to 36% (46). From 2002 to 2011, the mortality rate associated with ovarian cancer decreased significantly by 2% per year among White women, 1.4% per year among Hispanic women, but remained unchanged among Black women (47).

Several studies have demonstrated that worse survival outcomes among the Black population results from barriers that impede access to quality care and standardized evidence-based surgical and adjuvant treatment (32, 36, 48). Although the incidence of ovarian cancer is higher among White women (12.8 new cases per 100,000) compared to Black women (9.8 new cases per 100,0000), Black women tend to present with more advanced stage ovarian cancer compared to White women (49, 50). Black women have a higher incidence of medical comorbidities compared to White women that may influence treatment decisions (51, 52). However, several studies evaluating large nationally representative databases have demonstrated that Black patients are less likely to receive treatment consistent with evidence-based guidelines and have worse survival outcomes even after accounting for stage and comorbidities (32, 36, 37, 53). Parham et al. found that Black patients were less likely to receive combined surgery and chemotherapy treatment (48). In an analysis of a state specific database, Bristow et al. found that compared to White patients, Black race was associated with a statistically significant and independent lower likelihood of hysterectomy, lymphadenectomy, bowel resection, and surgery by a high-volume surgeon (54). Goff et al. also found that Black and Hispanic patients were also less likely to receive comprehensive staging compared to White patients (14). A SEER analysis by Wright et al. demonstrated delayed administration of adjuvant chemotherapy in Black patients, which was associated with an increased mortality rate (55). Importantly, the difference in survival outcomes among races is reduced or eliminated after accounting for access issues, socioeconomic status, stage, and treatment (4). The similarity in survival outcomes is highlighted in several GOG clinical trials where Black and White women receive similar treatments (56, 57). After review of available literature, it appears that equal treatment yields equivalent survival outcomes for both Black and White patients with ovarian cancer (4).

## Endometrial Cancer

Endometrial cancer is the most common gynecologic cancer in the US accounting for 54,870 new cases and 10,170 deaths in 2015 (31). For all stages, the 5-year survival rate is 82%, 95% for local disease, 68% for regional disease, and 18% for distant metastatic disease (31). White women had the highest incidence of endometrial cancer compared to other ethnic groups (24.8 per 100,000); however, the mortality rate is twice as high for Black women (7.3 per 100,000 vs. 3.9 per 100,000) (58). The 5-year survival for White women from 2004 to 2010 was 85% compared to 65% for Black women over the same time period (46). Similar to ovarian cancer, several studies have attributed this difference in survival to inequalities in access to care, adherence to evidence-based treatment guidelines, and socioeconomic barriers (59, 60). Unlike ovarian cancer, there may be inherent differences in tumor biology between White and Black patients with endometrial cancer as equal treatment has not correlated with equal outcomes (4). Black patients tend to be diagnosed at higher stages, with higher grade lesions, and high-risk histologies (61–65). Although worse tumor characteristics are associated with worse overall survival, after accounting for all histopathologic and sociodemographic factors, several large database analyses demonstrated worse survival associated with Black race (66–70). Black patients are less likely to be treated for advanced disease and less likely to undergo surgery (62, 71–73). However, Black women are more likely to be treated at high volume institutions with high volume specialized surgeons (74). When staging lymphadenectomy was performed, there were similar rates between Blacks and Whites (64). Other studies have demonstrated similar use of adjuvant chemotherapy and radiotherapy (73). Despite similar treatment, worse overall survival persists among Black women with endometrial cancer. In a GOG randomized clinical trial for advanced and recurrent endometrial cancer, Black women had a 26% greater chance of death compared to White women despite similar surgical and chemotherapeutic treatments after controlling for prognostic factors (75). Several studies have evaluated molecular differences in tumors from Black and White women in effort to identify why Black women have poorer prognosis relative to White women. These studies have primarily focused on p53 mutations, HER2/neu expression, and PTEN mutations. Mutations in tumor suppressor gene p53 have been associated with non-endometrioid histology, high grade tumors, advanced stage at presentation, and poorer overall survival (76). Clifford et al. demonstrated that Black women with stage I tumors were three times more likely to overexpress mutant p53, associated with worse survival and higher recurrence rates (77). Santin et al. demonstrated threefold higher HER2/neu expression in Black patients with serous endometrial cancer than in White patients with the same histology. The investigators concluded that overexpression of Her2/neu was an independent variable associated with poorer survival outcomes (78). Maxwell et al. demonstrated fewer PTEN mutations, associated with better outcomes and endometrioid histology, among Black patients compared to White patients (79). Further genetic and molecular studies need to be performed to further elucidate the causes of worse overall prognosis of Black patients with endometrial cancer.

## Cervical Cancer

Cervical cancer is the fourth most common cancer in the world. In the US, with the success of cervical cancer screening, the annual incidence is 12,900 with 4,100 deaths in 2015 (31). In 2015, the incidence of cervical cancer for Blacks was 11.4 per 100,000, 13.8 per 100,000 for Hispanics, and 8.5 per 100,000 for non-Hispanic Whites. The mortality rate was 4.9 per 100,000 for Blacks, 3.3 per 100,000 for Hispanics, and 2.3 per 100,000 non-Hispanic Whites (80). The overall 5-year survival for cervical cancer from 2004 to 2010 among White women was 71% compared to 62% in Black women (31). Interestingly, although the mortality rate is higher for Hispanics compared to non-Hispanic Whites, the 5-year survival for cervical cancer is 75% among Hispanic women compared to 71% for non-Hispanic Whites (41). Disparities in cervical cancer incidence and mortality are a direct reflection of unequal access to prevention, screening, and ultimate treatment. Data from the National Immunization Survey demonstrated a lower rate of HPV vaccination among Black and Hispanic adolescent girls compared to White adolescent girls (81). Although Black adolescents were more likely to initiate HPV immunization, they were less likely to complete the three-dose injection series (82, 83). Congress passed the Breast and Cervical Cancer Mortality Prevention Act of 1990, which allowed low-income, uninsured, and underinsured women to gain access to breast and cervical cancer screening and diagnostic services. Overall, 83% of women who have not had hysterectomies reported having a Pap smear in the prior 3 years, including 85% of Black women, 83.5% of White women, and 79% of Hispanic women (84). Despite the relative success with initiating screening, differences in follow-up from abnormal cervical cytology remains an issue, with Black women the most likely to be lost to follow-up (85). Consequently, Black women were more likely to present with more advanced disease than White women (31).

Additionally, treatment differences related to race have been shown to play a role in outcome disparities. Black women were less likely to receive a radical hysterectomy than White women for early stage cervical cancer (86) and were less likely to receive intra-cavity radiation therapy for locally advanced disease (87). Farley et al. demonstrated that in an equal access environment with identical treatment for cervical cancer between White and Black patients, there was equivalent 5- and 10-year survival data between races, reinforcing the idea that equal care results in equal survival outcomes in cervical cancer (88).

## Conclusion

Health care disparities in the incidence and outcome of gynecologic cancers persist and, in some cases, are worsening. The explanation for these disparities is complex and involves racial, economic, geographic, and biologic factors that influence treatment and survival. Much of the information available outlining these disparities have focused on disparities between Black and White women, with limited studies available regarding other minority populations. Additionally, as most of the studies investigating health disparities evaluated large nationally representative databases with limited detailed clinical information, it is not possible to account for other confounding factors that may have influenced treatment decisions or deviations from evidence-based guidelines. Despite diagnostic and therapeutic advances that have resulted in improved survival among American women in general, significant barriers exist in providing optimal care to millions of women in the US with gynecologic cancer. While not all factors involved in healthcare disparities are modifiable, identification and elimination of those that are must be a considered a top priority in a country that considers access to quality healthcare a basic human right.

## Author Contributions

SC was responsible for conducting the literature search and formulating the content of the manuscript. DG, TC, and KH were responsible for editing and adjusting content within the manuscript.

## Conflict of Interest Statement

The authors declare that the research was conducted in the absence of any commercial or financial relationships that could be construed as a potential conflict of interest. The reviewer, OB, declared a shared affiliation, though no other collaboration, with one of the authors, KH, to the handling Editor, who ensured that the process nevertheless met the standards of a fair and objective review.
